# Cyclooxygenase-1 null mice show reduced neuroinflammation in response to β-amyloid

**DOI:** 10.18632/aging.100021

**Published:** 2009-02-11

**Authors:** Sang-Ho Choi, Francesca Bosetti

**Affiliations:** Molecular Neuroscience Unit, Brain Physiology and Metabolism Section, National Institute on Aging, National Institutes of Health, Bethesda, Maryland 20892, USA

**Keywords:** cyclooxygenase, microglia, neuroinflammation, oxidative stress, neurotoxicity

## Abstract

Several independent epidemiological studies indicate that chronic use of non-steroidal anti-inflammatory drugs can reduce the risk of developing Alzheimer's disease (AD), supporting the inflammatory cascade hypothesis. Although the first clinical trial with indomethacin, a preferential cyclooxygenase (COX)-1 inhibitor, showed beneficial effects, subsequent large clinical trials, mostly using COX-2 inhibitors, failed to show any beneficial effect in AD patients with mild to severe cognitive impairment. These combined data suggest that either an early treatment is crucial to stop the mechanisms underlying the disease before the onset of the symptoms, or that preferential COX-1 inhibition, rather than COX-2, is beneficial. Therefore, a full understanding of the physiological, pathological, and/or neuroprotective role of COX isoforms may help to develop better therapeutic strategies for the prevention or treatment of AD. In this study, we examined the effect of COX-1 genetic deletion on the inflammatory response and neurodegeneration induced by β-amyloid. β-amyloid (Aβ_1-42_) was centrally injected in the lateral ventricle of *COX-1*-deficient (*COX-1^-/-^*) and their respective wild-type (WT) mice. In *COX-1^-/-^* mice, Aβ_1-42_-induced inflammatory response and neuronal damage were attenuated compared to WT mice, as shown by Fluoro-Jade B and nitrotyrosine staining. These results indicate that inhibition of COX-1 activity may be valid therapeutic strategy to reduce brain inflammatory response and neurodegeneration.

## Introduction

Alzheimer's disease (AD) is an aging-related progressive neurodegenerative disease, characterized by massive neuronal and synaptic loss, accompanied by neuropathological changes, such as neurofibrillary tangles and senile plaques, in the hippocampus, neocortex, and subcortical structures [1]. The senile plaques are primarily composed of amyloid beta peptide (Aβ), which is a 40-42 amino acid peptide fragment of the amyloid protein precursor. However, the mechanism by which Aβ causes neuronal injury and cognitive impairment is unclear. AD is also thought to have a local, non-immune mediated neuroinflammatory component with clusters of  activated  microglia, increased inflammatory proteins (complement factors, acute-phase protein, pro-inflammatory cytokines) [2-4], and increased COX-1-expressing microglia surrounding amyloid plaques [2]. Changes in COX-2 expression in AD are discrepant and seem to depend on the stage of the disease, with an upregulation of COX-2 in early AD, and a downregulation in advanced AD stages, which also correlate with PGE_2_ levels in the CSF, which are increased in probable AD patients and decrease with the progression of the disease [5,6]. Several independent epidemiological studies have shown that early use of non steroidal anti-inflammatory drugs (NSAIDs), which inhibit COX activity, significantly reduces the risk of developing AD later in life suggesting that inflammation is critical for the progression of the disease [7-13]. However, although a 6-month, double-blinded, placebo-controlled study with indomethacin, a preferential COX-1 inhibitor, appeared to protect AD patients from cognitive decline [14], subsequent large-scale randomized clinical trials, mostly with selective COX-2 inhibitors, did not show any beneficial effects in AD patients with mild to severe symptoms [15-18]. Supporting these clinical data, indomethacin, but not the COX-2 selective nimesulide, significantly reduced levels of Aβ in the hippocampus and cortex of transgenic mouse models of AD [19]. While the clinical data seem to rule out a protective effect of selective COX-2 inhibition in AD, it is still unclear whether COX-2 inhibitors can improve the pathology in animal models of AD. For instance, COX-2 inhibition blocks Aβ-mediated suppression of long-term potentiation and memory function, independently of reductions in Aβ_1-42 _or in inflammation [20]. However, the selective COX-2 inhibitor celecoxib has been shown to increase Aβ levels [21,22], and in a model of acute inflammation, both genetic deletion and pharmacological inhibition of COX-2 worsen the neuroinflammatory response to lipopolysaccharide (LPS) [23]. These combined data suggest that either NSAIDs have rather a preventive than a therapeutic effect or that preferential COX-1 inhibition is a better therapeutic approach than selective targeting COX-2, or that the beneficial effects are due to COX-independent effects of NSAIDs. In particular, ibuprofen, flurbiprofen, and diclofenac have been shown to reduce serum Aβ_1-42_ levels, a major component of senile plaques in AD [24-28]. However, a recent report from a pooled dataset from six prospective studies indicated that NSAIDs use reduced the risk of AD without any apparent advantage for the subset of NSAIDs shown to selectively lowering Aβ_1-42_[29]. While COX-1 and COX-2 are both differentially expressed in different stages of AD pathology, their specific roles in the pathogenesis of AD is unclear. Therefore, a full understanding of the physiological, pathological, and/or neuroprotective role of COX isoforms may help to develop better therapeutic strategies for the prevention or treatment of AD.

Partial reproduction of AD neuropathology and cognitive deficits has been achieved with pharma-cological and genetic approaches. Most injection models use synthetic peptide Aβ_1-40_ or Aβ_1-42_, which are analogous to peptides found in neuritic plaques in AD patients [30]. Mice with a null mutation of COX gene have been a useful tool for investigating the role of each COX isoform in both physiological and pathological conditions in the CNS by overcoming the complexity of dosing paradigm, duration of treatment, and possible unspecific inhibition of both COX isoform [31]. In this study, we assessed the effect of intracerebroventricular (i.c.v.) injection of Aβ_1-42_ on acute neuroinflammatory response in *COX-1*-deficient (*COX-1^-/-^*) mice and their respective wild-type mice (WT) controls. We showed that *COX-1^-/-^* mice are more resistant than WT mice to Aβ_1-42_-induced neuronal death and exhibit a marked reduction in the inflammatory response.

## Results

### The inflammatory response is reduced in *COX-1^-/-^* mice after Aβ_1-42_ injection

**Figure 1.**
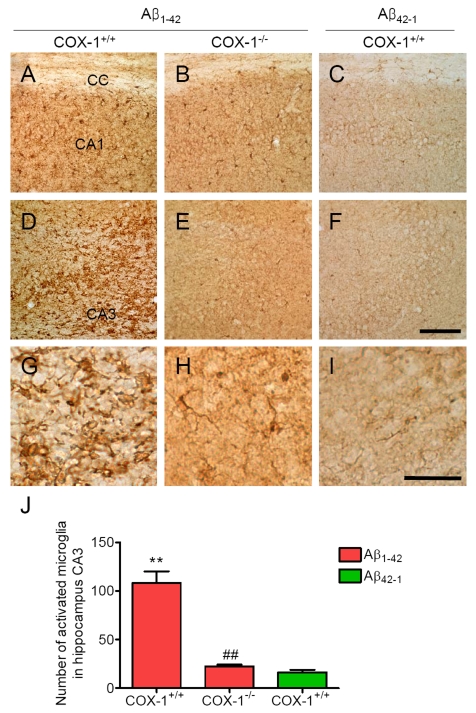
Increased microglial activation in the hippocampus 7 d after Aβ _1-42_ administration. Representative photomicrographs of the CA1 and CA3 of the hippocampus from WT mice (**A, D**) injected with Aβ_1-42_ that shows numerous activated microglia with short, less-ramified processes, perikaryal hypertrophy, and amoeboid appearance (**G**). CA1 and CA3 areas of the hippocampus from Aβ_1-42_-injected *COX-1^-/-^* mice (**B, E**) show many resting microglia with ramified morphology (H). Scale bar: A-F, 100 μm; G-I, 50 μm. (**J**) Comparison of the number of activated microglia from the CA3 area. Mean ± SEM (*n* = 3-4 per group); ^**^P < 0.01 compared with the Aβ_42-1_-injected WT mice; ^##^*P* < 0.01 compared with the Aβ_1-42_-injected WT mice.

Aβ_1-42_ or the control reverse peptide Aβ_42-1_ was unilaterally injected into the lateral ventricle, as reported [32-35]. Seven days later, brains were removed and coronal sections were processed for immunohistochemistry. We assessed microglial activation in the brain using IBA-1 as a microglial marker. Aβ_1-42_ administration caused a robust inflammatory response within the CA1 and CA3 areas of the hippocampus of WT mice characterized primarily by the presence of activated microglia (Figure 1A, D, J). Intense IBA-1-immunoreactive microglia with enhanced staining intensity, retracted processes, perikaryal hypertrophy, and amoeboid appearance were observed in the CA3 area of hippocampus of WT mice (Figure 1G). In *COX-1^-/-^* mice, IBA-1-immunreactive microglia retained a resting morphology with specifically small cell bodies, thin, and ramified processes (Figure 1B, E, H, J). In reverse peptide Aβ_42-1_-injected mice, only a few faintly IBA-1-immunoreactive microglia were observed in the hippocampus (Figure 1C, F, I, J). Staining with CD11b, another marker for microglia gave results similar to that of IBA-1 (data not shown).

**Figure 2.**
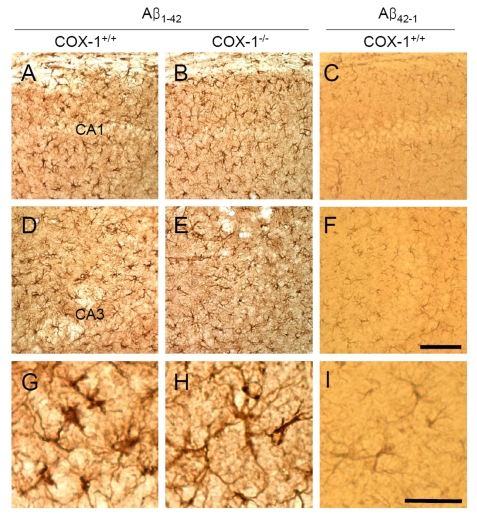
Increased astrocytic activation in the hippocampus 7 d after Aβ _1-42_ administration. Representative photomicrographs of the CA1 and CA3 of the hippocampus from WT mice (**A, D, G**) injected with Aβ_1-42_ that shows numerous robustly GFAP-immunoreactive astrocytes compared with Aβ_1-42_-injected *COX-1^-/-^* mice (**B, E, H**). Scale bar: A-F, 100 μm; G-I, 50 μm.

We then assessed astrocytes immunoreactivity by staining the brain of WT and *COX-1^-/-^* mice with the astrocytic marker glial fibrillary acidic protein (GFAP). GFAP-immunoreactive astrocytes in response to Aβ_1-42_ injection were markedly attenuated in the brain of *COX-1^-/-^* mice (Figure 2B, E, H) compared to WT mice (Figure 2A, D, G). These results indicate that Aβ_1-42_ administration induced less severe glial cell activation in *COX-1^-/-^* mice compared to WT mice.

### COX-1 deficiency leads to reduced neuronal damage following Aβ_1-42_ injection

**Figure 3.**
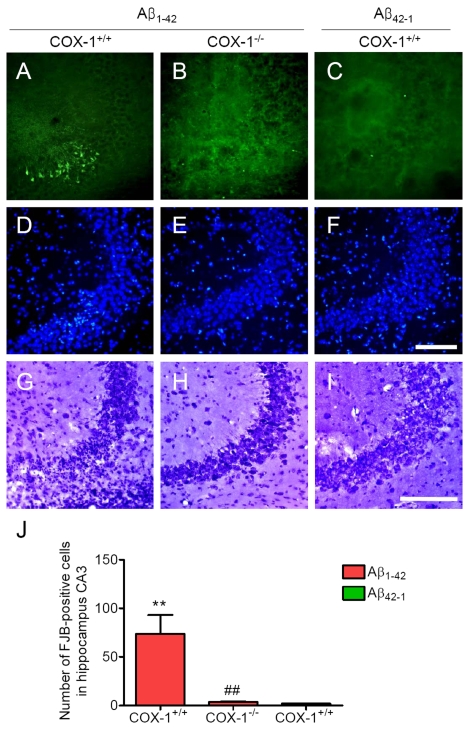
Increased degenerating neurons in the hippocampus 7 d after. **Aβ_1-42_ administration.** (**A-C**) Representative photomicrographs of the CA3 of the hippocampus from WT mice (**A**) injected with Aβ_1-42_ that shows numerous FJB-positive cells compared with Aβ_1-42_-injected *COX-1^-/-^* mice (**B**). Representative photomicro-graphs of DAPI (**D-F**) and Nissl staining (**G-I**) in the CA3 of hippocampus from Aβ_1-42_-injected WT (**D, G**) and* COX-1^-/-^*mice (**E, H**). Scale bar: A-I, 100 μm. (**J**) Comparison of the number of FJB-positive cells from the CA3 area. Mean ± SEM (*n* = 3-4 per group); ^**^P < 0.01 compared with the Aβ_42-1_-injected WT mice; ^##^*P* < 0.01 compared with the Aβ_1-42_-injected WT mice.

We next assessed neuronal damage in the brain using the fluorescent marker Fluoro-Jade B (FJB), which selectively labels injured neurons [36,37]. Aβ_1-42_ administration caused a significant neuronal damage, characterized by the presence of FJB-positive neurons within the CA3 areas of hippocampus of WT mice (Figure 3A, J). In contrast, Aβ_1-42_-injected *COX-1^-/-^* mice showed few scattered FJB-positive neurons in the CA3 of hippocampus (Figure 3B, J). In same sections stained with DAPI or adjacent sections stained with cresyl violet, a similar distribution of neuronal loss and gliosis was found in the CA3 areas of hippocampus in Aβ_1-42_-injected WT mice (Figure 3D, G). FJB and Nissl staining showed that hippocampal CA3 neurons in *COX-1^-/-^* mice were better preserved than in WT mice (Figure 3E, H). These results indicate that Aβ_1-42_ administration induced less severe neuronal damage in *COX-1^-/-^* mice compared to WT mice.

### *COX-1^-/-^* mice exhibit reduced oxidative damage following Aβ_1-42_ administration

**Figure 4.**
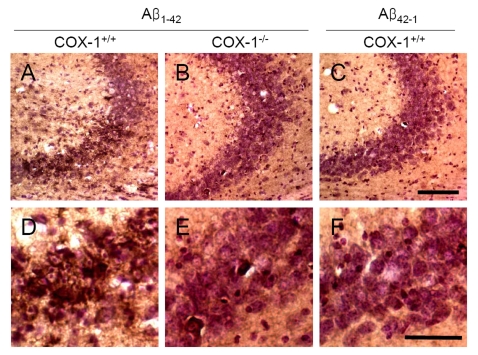
Increased oxidative damage in the hippocampus 7 d after Aβ _1-42_ administration. Representative photomicrographs of the CA1 and CA3 of the hippocampus from WT mice (**A, D**) injected with Aβ_1-42_ that show numerous robustly nitrotyrosine-immunoreactive cells compared with Aβ_1-42_-injected *COX-1^-/-^* mice (**B, E**). Scale bar: A-C, 100 μm; D-F, 50 μm.

An important component of Aβ_1-42_-induced neurotoxic process is mediated by oxidative damage [38], which can be evaluated by assessing protein carbonyls and nitrotyrosine levels [39]. To determine whether oxidative damage is involved in the process of Aβ_1-42_-induced neurotoxic process, we investigated oxidized amino acid, nitrotyrosine levels using sections adjacent to those used for FJB staining. We found an increase in nitrotyrosine-immunoreactive cells in the brain of WT mice (Figure 4A, D), which was markedly attenuated in the brain of *COX-1^-/-^* mice (Figure 4B, E). These results indicate that Aβ_1-42_ administration induced less severe oxidative damage in *COX-1^-/-^* mice compared to WT mice.

### PG generation is reduced in Aβ_1-42_-injected *COX-1^-/-^* mice

**Figure 5.**
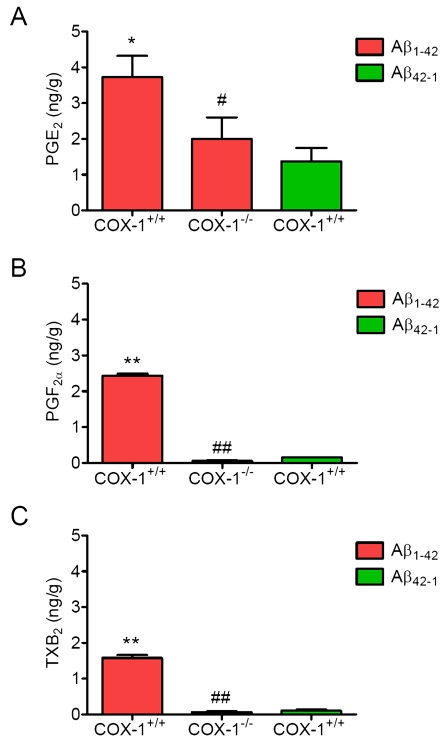
Effects of COX-1 deficiency on PG production 24 h after Aβ _1-42_ administration. Aβ_1-42_-injected WT mice show significantly more PGE_2_ (**A**), PGF_2α_ (**B**), and TXB_2_ levels (**C**) than *COX-1^-/-^* mice. Mean ± SEM (*n* = 3-4 per group); ^*^P < 0.05, ^**^P < 0.01 compared with the Aβ_42-1_-injected WT mice; ^#^*P* < 0.05, ^##^*P* < 0.01 compared with the Aβ_1-42_-injected WT mice.

To determine the contribution of COX-1 to PG production after Aβ_1-42_ injection, we measured the levels of PGE_2_, PGF_2α_, and TXB_2_ 24 h after Aβ_1-42_ administration. We observed significant reduction in levels of PGE_2_ (Figure 5A), PGF_2α_ (Figure 5B), and TXB_2_ (Figure 5C) inAβ_1-42_-injected* COX-1^-/-^* mice.

These results suggest that the reduced levels of PGE_2_, PGF_2α_, and TXB_2_ in *COX-1^-/-^* mice could contribute, in part, to the observed differences in glial and neuronal response to Aβ_1-42_ administration.

## Discussion

In this study, genetic deletion of *COX-1* led to a decrease in the inflammatory response and neuronal damage in response to Aβ_1-42_, and this effect was associated with alteration of PG production. We show that Aβ_1-42_-induced oxidative damage and degenerating neurons, as well as glial activation, were less severe in *COX-1^-/-^* mice compared to WT mice. These data suggest that COX-1 facilitates activation of glial cells and supports inflammatory processes and oxidative stress that evolve in neuronal damage, and support previous data from our lab showing that *COX-1^-/-^* mice have a decreased inflammatory response, oxidative stress and neuronal damage after central injection of LPS [37].

Glial cell activation, in turn, results in enhanced production of a variety of proinflammatory and oxidative mediators, including cytokines, chemokines, and reactive oxygen/nitrogen species [40-42]. Oxidative stress has been recognized to play an important role in the pathogenesis of AD and linked to the presence of Aβ by the finding of several characteristics, such as enhanced protein, DNA oxidation, and lipid peroxidation in specific regions of the postmortem brain [43-48]. A previous study suggested that oxidative DNA damage reduces the expression of highly vulnerable genes involved in neuronal survival and learning memory, initiating a program of brain aging that starts early in adult life [49]. In addition, lipid peroxidation leads to a reduction in membrane fluidity, alteration of membrane-bounded protein, receptors, and ion channels, and generation of Aβ that induces more oxidative stress and calcium influx that induces glutamate excitotoxicity and cell death [50,51]. The abundant polyunsaturated lipid content, high oxygen consumption, high metal ion concentration, and low regenerative capacity, as well as relatively low antioxidant levels compared with other tissues make brain highly susceptible to oxidative damage [49,52]. In addition, oxidative stress differentially affects brain regions, as levels of peroxidizable unsaturated lipids and antioxidant enzymes, and membrane-bound protein differ between brain regions. In this regard, continuous icv infusion of Aβ_1-42_ results in a significant reduction of endogenous antioxidant systems, including Mn-superoxide dismutase (Mn-SOD), glutathione, glutathione peroxidase, and glutathione-S-transferase-π in the hippocampus, cortex, substantia nigra, and thalamus [53]. Importantly, these alterations of each antioxidant enzyme were not uniform, but rather specific in a brain region-dependent manner (e.g. Mn-SOD in CA3), indicating a heterogenous susceptibility to the Aβ_1-42_-induced oxidative stress.

Our results show that a single injection of Aβ_1-42_ resulted in a similar spatial distribution of reactive glial cells, nitrotyrosine, and degenerating neurons in the CA3 of hippocampus, suggesting the possibility that glial cell-derived reactive oxygen/nitrogen species may be involved in the impaired neuronal function, which has been described in this model [32,33,54,55]. Indeed, several studies have shown that pretreatment with antioxidants or minocycline, a tetracycline derivative with anti-inflammatory and neuroprotective properties, tend to ameliorate the Aβ_1-42_-induced oxidative damage and behavioral deficits [32,33,56]. Although, variable in terms of the injected Aβ peptide sequences, injection methods, and employed behavioral tests, previous studies have consistently shown the occurrence of behavioral deficits related to memory impairment after intracerebral injection of Aβ peptide [32,33,57-59]. Therefore, Aβ injection is a useful *in vivo* model for Aβ toxicity, which is an important component in the progression of AD.

Gene deletion of *COX-1* decreased glial cell activation and attenuated nitrotyrosine induction. The decreased oxidative damage in *COX-1^-/-^* mice suggests that *COX-1* deletion can reduce the activity of free-radical generating enzymes such as inducible nitric oxide (iNOS), NADPH oxidase, and myeloperoxidase (MPO). These data are consistent with recent observations that genetic deletion of *COX-1* significantly reduces LPS-induced expression of both superoxide (O_2_^-^) and NO-forming enzymes and thus subsequently attenuates the levels of nitrotyrosine and protein carbonyls, which are considered as biomarkers of oxidative stress [37]. Although, the precise mechanism(s) by which COX-1 regulates free radical-generating enzymes in inflammatory cascade have not been clearly established, it is possible that because of its predominant localization in microglia, COX-1 can modulate the induction of O_2_^-^, as well as NO, from NADPH oxidase and iNOS, which, in turn, can enhance the production of more potent free radicals such as peroxynitrite (ONOO^-^). In addition, O_2_^-^ and NO act as potent cell signaling molecules and amplify production of TNF-α and PGE_2_ by upregulation of COX-2 [60]. These initial effects combined with the activation of seconddary signaling cascades activate a robust immune response that consequently causes neuronal damage and death.

The results from epidemiological data indicating that NSAIDs are effective in preventing or delaying the onset of AD combined with the failure of COX-2 selective inhibitors in clinical trials in AD patients with moderate to severe AD suggest that either an early treatment is crucial to stop the mechanisms underlying the disease before the onset of the symptoms or that COX-2 selective inhibitors are not effective in delaying the progression of AD. In this regard, an intriguing hypothesis is that the protective effects of NSAIDs may be related to COX-1 rather than COX-2 inhibition. Supporting this hypothesis, COX-1 selective inhibitors (SC-560 and valeryl salicylate), but not COX-2 selective inhibitors (SC-236 and DuP-697), reduce Aβ_1-42_-induced PGs production and neurotoxicity in postmortem human microglia and in murine cortical neurons [61,62]. Furthermore, a small double blind, placebo-controlled study with indomethacin, a preferential COX-1 inhibitor [63], appeared to protect mild to moderately impaired AD patients from cognitive decline [14]. Interestingly, COX-1 is prominently expressed by microglia in rodent and human brain [2,4] and appears to be increased in AD brain [2]. Double immunostaining for Aβ and COX-1 indicates clustering of COX-1 positive microglia with classicaland neuritic plaques, although there is no indication that COX-1 is upregulated in activated microglia [64]. However, LPS-induced PGE_2_ secretion can be reduced by COX-1 genetic deletion and by COX-1 selective inhibitors [37,61,65], suggesting that it is dependent on the constitutive COX-1 activity. In contrast, COX-2 has not been detected in microglia and astrocytes in AD [66]. These combined data suggest that COX-2 may not be the exclusive COX isoform responsible for patho-physiological consequences in neurodegenerative diseases, especially in AD, but that COX-1 also plays a critical role in the process of neuroinflammation and neurodegeneration.

In summary, we show that COX-1 facilitates activation of glial cells and supports inflammatory processes and that genetic deletion of COX-1 significantly attenuates the oxidative stress and neuronal damage in response to Aβ_1-42_. This effect may be due to the predominant localization of COX-1 in microglial cells, where, through its prostaglandin products contributes to the neuroinflammatory cascade of events that ultimately lead to neuronal damage or death. Therefore, COX-1 may represent a viable therapeutic target to treat neuroinflammation and neurodegeneration.

## Materials and methods

Animals and stereotaxic A β _1-42_ administration. Three-month-old male homozygous *COX-1^-/-^* and their WT mice (*COX-1^+/+^*) on a C57BL/6-129/Ola genetic background were used [67]. Mice were received at our animal facility at 6 weeks of age from a NIEHS colony maintained by Taconic Farms (Germantown, NY) with heterozygous by heterozygous breedings for greater than 35 generations. In order to prevent the inclusion of strain or genetic background confounders between *COX* null and wild type mice, all of the mice used in this study were progeny derived from heterozygous by heterozygous mating and therefore all contained the same strain and genetic background [67,68]. The mice were housed at 25°C in our animal facility with a 12 h light/dark cyclewith free access to food and water. All animal procedures were approved by the National Institutes of Health (NIH) Animal Care and Use Committee in accordance with NIH guidelines on the care and use of laboratory animals. Aβ_1-42 _and reverse peptide Aβ_42-1_ (American Peptide, Sunnyvale, CA) were reconstituted in phosphate-buffered saline (pH 7.4) and aggregated by incubation at 37°C for 4 days before use as described previously [69]. Aβ_1-42 _and Aβ_42-1_ (400 pmol per mouse) were administered intracerebroventricularly (i.c.v) into the lateral ventricle using a 10 μl syringe with a fine needle (World Precision Instruments, Sarasota, FL) and a syringe pump (Stoelting, Wood Dale, IL) at a rate of 1 μl/min. The dose of Aβ_1-42 _and Aβ_42-1_ was selected based on previous studies [32-35]. The coordinates for the stereotaxic injections were -2.3mm dorsal/ventral, -1.0 mm lateral, and -0.5 mmanterior/posterior from the bregma [70].

Tissue preparation and histology. Mice were transcardially perfused withsaline followed by 4% paraformaldehyde. Brains were postfixedovernight in the same medium and placed in 30% sucrose, before sectioning (30 μm). Immunohistochemistry and double immunofluorescence were performed as described previously [71]. Rabbit anti-IBA-1 (1:500; Wako), mouse anti-GFAP (1:200; Sigma-Aldrich), and mouse anti-nitrotyrosine (1:100; Chemicon, Temecula, CA) were used as primary antibodies. The slides were visualized by brightfield microscopy (Olympus) and digitally photographed. FJB, a fluorochrome for the sensitive histochemicallocalization of neuronal degeneration, was used to identifydegenerating neurons [72]. Brainsections were mounted on gelatin-coated slides and completelydried. Then sections were rehydrated through graded concentrationsof alcohol (100, 70, and 50%; 1 min each), and rinsed for 1 min in distillated water. The slides were incubated in a solution of 0.06% potassium permanganate for 20 min, rinsedin distilled water for 1 min, and transferred to FJB (Histochem, Jefferson, AR) stainingsolution (0.001% FJB/0.1% acetic acid) for 20 min. The slides were thereafter rinsed three times in distilled water and air dried then immersedin xylene and coverslipped with mounting media. The slides were visualized by fluorescent microscopy (Olympus) and digitally photographed. Because the FJB staining was obvious on digital imaging, the number of FJB-positive cells per section was quantified as described previously [73]. The number of microglia per section was quantified by counting the number of IBA-1-stained cell bodies within 0.3 mm^2^ area of the CA3. For each measurement, two blinded independent investigators counted 3-4 brains per group, 3 sections per brain.

Measurement of prostanoids. Prostanoids were purified from the lipid extract as previously described [74] and levels were determined using specific enzyme immunoassay (EIA) kits, PGE_2_, PGF_2α_, and TXB_2_, (Oxford Biomedical, Oxford, MI).

*Statistics. *All data are expressed as mean ± SEM. Statistical significance was assessed with one-way analysis of variance (ANOVA) followed by Bonferroni's post hoc test using GraphPad Prism version 4.00 (GraphPad Software, San Diego, CA). Significance was taken at *P *< 0.05.
